# Supplementary material for: Identification of Novel Loci and Candidate Genes for Cucumber Downy Mildew Resistance Using GWAS
Source: Plants (Basel). 2020 Nov 27;9(12):1659. doi: 10.3390/plants9121659 (PMC7768435; doi:10.3390/plants9121659)
Supplement: Supplementary file 1 [file plants-09-01659-s001.zip › Supplementary File/Fig S1-S2.pdf]

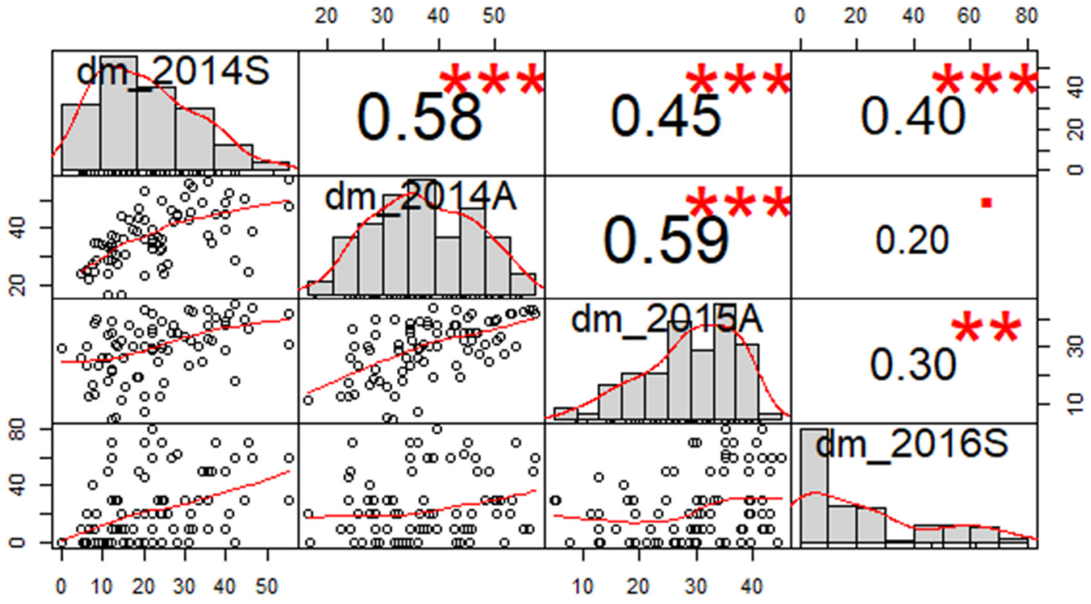

Figure S1. Frequency distribution and Spearman rank correlations of mean disease index of the CG lines in four experiments, dm_2014S, dm_2014A, dm_2015A, and dm_2016S. ** and *** indicate significance at $p < 0.001$ and $p < 0.01$, respectively.

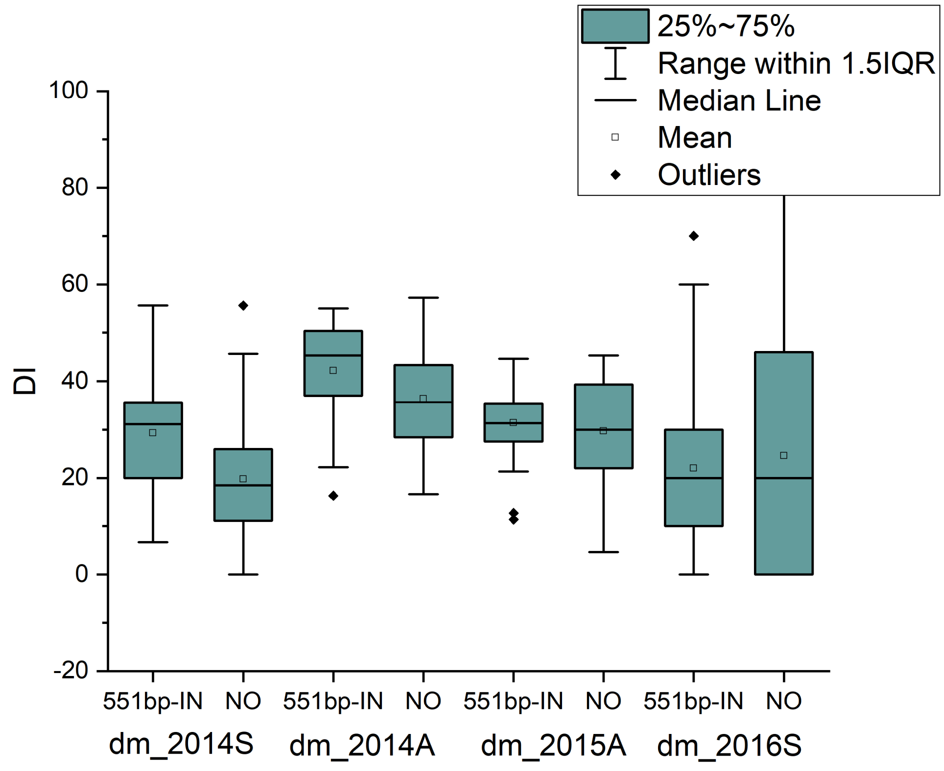

Figure S2. Disease index (DI) of four experiments for the CG lines with (551bp-IN) and without (NO) the 551-bp insertion in the RLK (*Receptor-like Kinase*) gene reported by Berg et al. 2020 [11].
