# Supplementary material for: Identification of Novel Loci and Candidate Genes for Cucumber Downy Mildew Resistance Using GWAS
Source: Plants (Basel). 2020 Nov 27;9(12):1659. doi: 10.3390/plants9121659 (PMC7768435; doi:10.3390/plants9121659)
Supplement: Supplementary file 1 [file plants-09-01659-s001.zip › Supplementary File/Supplementary file 4_SNPs and overlap with other study.pdf]

**Supplementary file 4.** Comparison of loci identified by GWAS with previously reported QTLs.

| Locus | SNP name | Chromosome | SNP Position | Flanking markers | Physical location (Mb) | Reference |
|---|---|---|---|---|---|---|
| dmG-1.1 | SNP31098 | 1 | 2,107,675 | UW044821<br>SSR05793 | 1.2<br>3.1 | Zhang et al. 2018 |
| dmG-1.2 | SNP187059 | 1 | 10,520,443 | UW084539<br>SSR16055 | 8.72<br>13.30 | Zhang et al. 2018 |
| dmG-1.3 | SNP293263 | 1 | 16,465,444 | Marker1_15701257<br>Marker1_26471365 | 15.70<br>26.47 | Wang et al.2018 |
| dmG-1.4 | SNP398090 | 1 | 21,761,223 | Marker1_15701257<br>Marker1_26471365 | 15.70<br>26.47 | Wang et al.2018 |
| dmG-2.1 | SNP639496 | 2 | 4,618,724 | | Novel | |
| dmG-2.2 | SNP730945 | 2 | 8,449,502 | SSR13532<br>SSR13105 | 5.64<br>10.28 | Win et al. 2017 |
| dmG-2.3 | SNP829346 | 2 | 12,989,299 | Marker2_7976333<br>Marker2_12537499 | 7.97<br>12.82 | Wang et al. 2018 |
| dmG-3.1 | SNP1139014 | 3 | 8,524,603 | SSR16264<br>SSR13312 | 9.15<br>10.25 | Win et al. 2017 |
| dmG-4.1 | SNP1793602 | 4 | 5,302,408 | SSR00012<br>SSR13159 | 4.11<br>20.10 | Cavagnaro et al.2011 |
| dmG-4.2 | SNP1938892 | 4 | 13,242,382 | SSR00012<br>SSR13159 | 4.11<br>20.10 | Cavagnaro et al.2011 |
| dmG-4.3 | SNP2065096 | 4 | 19,027,366 | SSR00012<br>SSR13159 | 4.11<br>20.10 | Cavagnaro et al.2011 |
| dmG-5.1 | SNP2452535 | 5 | 18,119,570 | Marker5_16446482<br>Marker5_18140438 | 16.44<br>18.14 | Wang et al.2018 |
| dmG-5.2 | SNP2531703 | 5 | 22,899,752 | SSR01498<br>InDel82 | 22.66<br>25.75 | Zhang et al. 2018 |
| dmG-6.1 | SNP13977656 | 6 | 13,977,656 | | 5.54<br>17.28 | Win et al. 2016 |
| dmG-6.2 | SNP3104337 | 6 | 25,297,012 | SSR01148<br>SSR05946 | 25.95<br>28.09 | Wang et al. 2016 |
| dmG-6.3 | SNP3158927 | 6 | 28,650,383 | SSR01148<br>SSR05946 | 25.95<br>28.09 | Wang et al. 2016 |
| dmG-7.1 | SNP3165616 | 7 | 84,900 | | Novel | |
| dmG-7.2 | SNP3517753 | 7 | 18,251,423 | SSR33278<br>SSR477 | 17.52<br>19.19 | Yoshioka et al. 2014 |
